# Can Tho oncology (Việt Nam) hospital radiotherapy department: A Snapshot of current and future practice

**DOI:** 10.1016/j.tipsro.2024.100292

**Published:** 2024-11-17

**Authors:** Tran Thanh Phong, Vo Van Kha, Le Thi Den

**Affiliations:** Can Tho Oncology Hospital, Viet Nam

**Keywords:** Can Tho oncology hospital, The Radiotherapy Department Mekong Delta area

## Abstract

Can Tho City Oncology Hospital was established on September 28, 2007, by the People’s Committee of Can Tho City. The Can Tho City Oncology Hospital has been in operation for 17 years and has over time become a reliable place for the provision of oncological patient services and treatment for Can Tho citizens and the Mekong Delta area. The Hospital includes approximately 400 patient beds, 9 clinical departments, 5 paraclinical departments (radiology, pathology etc), and 8 functional rooms (customer care epartment, admissions, discharge office, finance and accounting division,). The Radiotherapy Department is a busy and important part of the Hospital and plays an important role in cancer patient treatment.

Introducing the current situation, challenges and future plans for the Radiotherapy Department in Can Tho Oncology Hospital, Việt Nam.

## Introduction

Cancer is one of world’s leading cause of death accounting for almost 10 million deaths in 2020 [1]. Cancer poses a number of challenges globally. More specifically, cancer incidence projections have identified a growth in numbers is expected in low and middle income countries (LMIC). These countries are significantly under prepared to confront the cancer challenge [2]. Equitable access to cancer treatment has never been so critical, particularly for LMICs [2].

The South-Eastern Asian country of Việt Nam is the 17th most populous country in the world with over 100 million in 2024. It is projected to hit its peak of 109 million by 2054 [3].It is well documented that Việt Nam has experienced a significant increase in cancer incidence, with many cancers diagnosed at later stages (70 %) [4]. Since most patients present with late stage disease, the availability of radiation therapy is an essential treatment option, however, many regions in Việt Nam lack adequate infrastructure [5]. With the existing healthcare costs and the high burden of noncommunicable diseases (NCDs) such as cancer, access to treatment has never been more important.

Can Tho City is the biggest city in Mekong Delta, located on the left bank of the Hau Giang River, approximately 145 km Southwest of Ho Chi Minh City (formerly Saigon). Its population is nearing 1.6 million. Currently, Can Tho City Oncology Hospital only has 1 Cobalt radiotherapy machine, which is not enough to meet the treatment needs of the people in the area.

[6] Can Tho City Oncology Hospital was established on September 28, 2007, by the People's Committee of Can Tho City. The Can Tho City Oncology Hospital has been in operation for 17 years and has over time become a reliable place for the provision of oncological patient services and treatment for Can Tho citizens and the Mekong Delta area. The Hospital includes approximately 400 patient beds, 9 clinical departments, 5 paraclinical departments (radiology, pathology etc), and 8 functional rooms (customer care department, admissions, discharge office, finance and accounting). The Radiotherapy Department is a busy and important part of the Hospital and plays an important role in cancer patient treatment. It was established in April 2010. Prior to its establishment the Can Tho and Mekong Delta residents who were afflicted with cancer, were required to travel to Ho Chi Minh city which is geographically located 145kms from the local area.

The most commonly treated types of cancers treated at the Can Tho Radiotherapy Department include head and neck, breast, colorectal, and cervical cancers. Additionally, other presenting cancers include prostate cancer, lymphoma and lung cancer. The department is significantly large and includes a total of 43 staff, including nine (9) radiation oncologists, eight (8) medical physicists, eight (8) radiation therapists and 18 oncology nurses. The departmental radiation oncology equipment includes a Cobalt^60^ for external beam radiotherapy and CT-based image treatment planning (3D-CRT) with Varian Eclipse Software. Additionally, brachytherapy treatments are common practice in the department and these are delivered using microSelectronic High Dose Rate After Loading Brachytherapy with Iridium^192^ using 3D treatment planning on Oncentra Software.

**Fig. 1:**
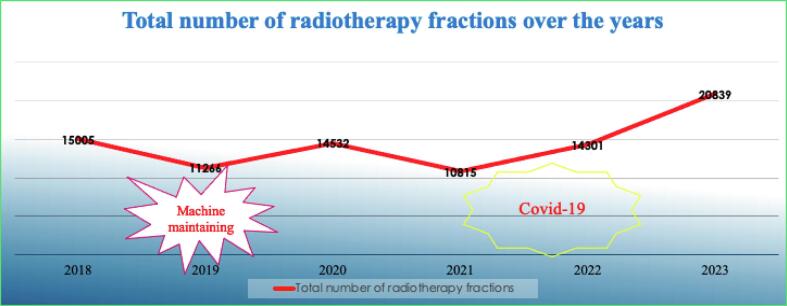
Total number of radiotherapy fractions 2022–2023.

The department’s simulation of patients is conducted using a 2D simulator primarily, however, the department also contains a 32 slice CT scanner used for diagnosis and at times, simulation. (See Fig. 1.).

There was a significant increase in radiotherapy fractions from 2022 to 2023. It shows that more and more patients come to Can Tho Hospital for treatment. It is necessary to invest in advanced techniques to serve treatment demands and improve treatment quality.

## Challenges

### Equipment

Whilst the Can Tho Oncology Hospital services a large population and treats ≥2,000 patients annually (∼800 out-patients, ∼1,200 in-patients) in the Radiotherapy Department, it is becoming apparent that the current equipment cannot sustain advanced technologies. These advanced practice techniques (IMRT, VMAT) have been reported to improve patient overall outcomes, efficiency in practice and a decrease in side effects generally. Currently, at Can Tho Oncology Hospital, the practitioners use a Cobalt^60^ to deliver treatments, equipment that cannot support advanced practice and techniques. Additionally, as time passes, the Cobalt^60^ source activity has decreased such that the patient treatment time is significantly increased, adding to a decrease in patient comfort, compliance and person-centred care. As a result, there is a large number of patients waiting for treatment, despite the Cobalt^60^ machine working 23 of the 24 h each day. Treatment is also delivered on the weekends to cover the patient load. Additionally, it is not unusual for patients to have to wait 2–3 months for treatment, resulting in poorer patient overall outcomes.

### Future plans

Overall, the hospital as it is, requires significant investment. The infrastructure and patient resources and services are limited and impact patient overall care and outcomes. As an example, it is not unusual for 2 patients to share a bed in the wards as a result of limited bed availability.

There are plans in place for a new hospital build. It is anticipated that the new hospital will include advanced radiotherapy techniques supported by 4 new accelerators (Linac and Truebeam), 2 High Dose Rate Brachytherapy machines, PET-CT, and a cyclotron. This will ultimately improve the patient waiting lists, increase treatment effectiveness and reduce current side effects. It will also support the further professional development of the clinical/medical staff.

We look forward to Can Tho Oncology Hospital becoming a specialised hospital providing breakthrough treatment options and state of the art equipment with which to deliver the best possible care for the people of Can Tho city and the center of the Mekong Delta area.

## Declaration of competing interest

The authors declare that they have no known competing financial interests or personal relationships that could have appeared to influence the work reported in this paper.
